# Essential conditions for the implementation of comprehensive school health to achieve changes in school culture and improvements in health behaviours of students

**DOI:** 10.1186/s12889-016-3787-1

**Published:** 2016-11-02

**Authors:** Kate E. Storey, Genevieve Montemurro, Jenn Flynn, Marg Schwartz, Erin Wright, Jill Osler, Paul J. Veugelers, Erica Roberts

**Affiliations:** 1 School of Public Health, University of Alberta, 3-50 University Terrace, 8303-112 Street, Edmonton, AB T6G 2T4 Canada; 2 The APPLE Schools Foundation, 3-50 University Terrace, 8303-112 Street, Edmonton, AB T6G 2T4 Canada; 3 Present address: 2210 2nd Street SW, Calgary, AB T2S 3C3 Canada

**Keywords:** Canada, Comprehensive school health, Essential conditions, Health promotion, Qualitative research

## Abstract

**Background:**

Comprehensive School Health (CSH) is an internationally recognized framework that holistically addresses school health by transforming the school culture. It has been shown to be effective in enhancing health behaviours among students while also improving educational outcomes. Despite this effectiveness, there is a need to focus on how CSH is implemented. Previous studies have attempted to uncover the conditions necessary for successful operationalization, but none have described them in relation to a proven best practice model of implementation that has demonstrated positive changes to school culture and improvements in health behaviours.

**Methods:**

The purpose of this research was to identify the essential conditions of CSH implementation utilizing secondary analysis of qualitative interview data, incorporating a multitude of stakeholder perspectives. This included inductive content analysis of teacher (*n* = 45), principal (*n* = 46), and school health facilitator (*n* = 34) viewpoints, all of whom were employed within successful CSH project schools in Alberta, Canada between 2008 and 2013.

**Results:**

Many themes were identified, here called conditions, that were divided into two categories: ‘core conditions’ (students as change agents, school-specific autonomy, demonstrated administrative leadership, dedicated champion to engage school staff, community support, evidence, professional development) and ‘contextual conditions’ (time, funding and project supports, readiness and prior community connectivity). Core conditions were defined as those conditions necessary for CSH to be successfully implemented, whereas contextual conditions had a great degree of influence on the ability for the core conditions to be obtained. Together, and in consideration of already established ‘process conditions’ developed by APPLE Schools (assess, vision, prioritize; develop and implement an action plan; monitor, evaluate, celebrate), these represent the essential conditions of successful CSH implementation.

**Conclusions:**

Overall, the present research contributes to the evidence-base of CSH implementation, ultimately helping to shape its optimization by providing school communities with a set of understandable essential conditions for CSH implementation. Such research is important as it helps to support and bolster the CSH framework that has been shown to improve the education, health, and well-being of school-aged children.

## Background

Worsening health behaviours of children including unhealthy eating, physical inactivity and sedentary behaviour pose a significant public health problem [1] as evidenced by the global childhood obesity epidemic [2], and the array of associated health complications and comorbidities [3]. These alarming trends emphasize the need for early intervention, through comprehensive health promotion and primary prevention strategies [4]. Schools act as ideal intervention settings as they can reach almost all children during critical periods of development [5]. Comprehensive School Health (CSH) is a framework that incorporates individual, interpersonal, community, and organizational factors, with direct and indirect influences on health. The pan-Canadian Joint Consortium for School Health (JCSH), frames CSH through four inter-related pillars: 1) teaching and learning; 2) social and physical environments; 3) healthy school policy; and 4) partnerships and services [6]. By targeting each of these areas, schools aim to transform their culture to support positive health practices [7]. CSH is synonymous with the term Health Promoting Schools and has been demonstrated as an effective model for school-based health promotion, positively influencing academic outcomes [8] as well as health behaviours in children [5, 9–12]. Despite this effectiveness, little research exists examining what specific conditions have contributed to this success, how these conditions work together to facilitate implementation and how best to implement this approach [13, 14].

Identifying the essential conditions of CSH implementation has been a recent focus by some in the field of CSH, in hopes that identification would facilitate the development and sustainability of healthy school communities, and promote rigour in evaluation [14, 15]. As such, some important conditions for CSH implementation have been identified such as stakeholder engagement, professional development, and resources [14, 16]. The way in which these (and other) fundamental conditions fit together and are operationalized by diverse stakeholders implementing CSH, however, remains less clear. Few studies have examined these implementation conditions within the context of projects that have proven effective in shifting a school culture [17] and improving health behaviours in children [9, 18], and therefore can claim successful implementation of CSH [19, 20]. We believe that it is important not only to know, in theory, what these conditions are, but also to understand how they work together to facilitate implementation within projects that have proven effective.

Thus, to address this gap the present study will use secondary analysis of previously published interview data from five studies regarding the implementation and sustainability of CSH [17, 21–24], to identify and operationalize the essential conditions of CSH implementation. Multiple stakeholder perspectives [25] from those working on the ground within successful CSH project schools in Alberta, Canada, will be included.

## Methods

### Setting

Within the present study, qualitative data were analyzed from two different CSH projects. These projects included the Alberta Project Promoting active Living and healthy Eating in Schools (APPLE Schools), and Healthy Schools–Healthy Future (HSHF). Both projects were implemented primarily in elementary schools throughout Alberta, Canada with the aim to improve healthy eating, active living, and positive social environments among children by increasing the capacity of the school community to support these healthy behaviours [26]. Each participating school received dedicated staff time in the form of a trained School Health Facilitator (SHF). The SHF actively engaged members of the school community to address barriers to healthy eating, active living, and positive social environments, working within each of the four pillars of CSH [26]. HSHF was funded by the Alberta government from January 2012 – June 2014 and was implemented in 17 school communities across rural Alberta, modelled after the success of APPLE Schools. APPLE Schools was initially funded by a private donation and is now funded by the federal government as well as private donations. APPLE Schools was originally launched within ten schools in Edmonton, Alberta in January of 2008 and now includes 50 school communities throughout central and northern Alberta.

The process in which APPLE Schools implements CSH has been recognized internationally as a best practice through both the Public Health Agency of Canada’s Best Practices Portal [19] and the National Cancer Institute’s Research-tested Intervention Programs [20]. The implementation process is iterative and collaborative and includes ‘process conditions’ which can be described as: 1. assess, vision and prioritize; 2. develop and implement an action plan; and 3. monitor, evaluate and celebrate [27]. APPLE Schools best practice documentation outlines the importance of establishing an APPLE Core or Wellness Committee to provide leadership in these ‘process conditions’ of CSH implementation [19, 20, 27]. Common responsibilities of the core committee include the ongoing involvement in professional development, the creation of the school health action plan, continuous involvement in evaluation and assessment, as well as representing the project inside and outside of the school. Committees are comprised of representatives from the school community including the SHF, administrator, students, teachers, school staff, parents, and community representatives [27].

### Data collection

While secondary analysis of qualitative data is an established practice, there are several considerations for its effective conduct when working across datasets. Issues concerning the relationship between primary and secondary analysis, context, and researcher reflexivity are important to consider [28]. For the purpose of this secondary analysis, we combined and examined data from five separate datasets to explore the essential conditions of CSH implementation. Each of the five studies were driven by community-based participatory research which has its roots in ethnography [29] and are therefore aligned methodologically. While the specific purpose of each study differed, their context was the same and the overarching objective for all was to explore implementation. In the literature, there are concerns regarding researcher presence and reflexivity and the privileged relationship the researcher holds with the primary data generated and how this affects secondary analysis [28]. However, as stated by Irwin and Winterton [28] “Primary analysts have a privileged relationship to the data they have generated, but do not necessarily have a privileged claim on the arguments which can be made from that data.” Given that the primary datasets were aligned methodologically, all participants were purposively sampled, the research objectives were comparable, the context of each study was similar, and the researchers were responsive and reflexive by revisiting themes, cross-checking findings and staying true to the iterative nature of the research; the authors felt that a secondary analysis was not only appropriate, but a strength. While each study included in this secondary analysis made an important contribution to the literature, the combination of the five studies into one dataset was unique and allowed for comparisons across stakeholder groups simultaneously. Therefore, by combining the datasets, trustworthiness in the data was actually improved [30]. The five datasets are described briefly below and will be referred to as DS1, DS2, DS3, DS4 and DS5 within the results.

### Primary datasets: school health facilitator, teacher, and principal interviews

APPLE SHFs were interviewed for two separate studies. The first study used structured interviews and explored SHF knowledge, skills, and attitudes immediately following training (*n* = 10, January 2008) and one year into implementation (*n* = 10, January 2009) and is described previously (DS1) [23]. The second study with SHFs utilized semi-structured interviews (*n* = 14, January-February 2011) and explored their perceptions of the process of CSH implementation including its facilitators and barriers (DS2) [24]. Focus groups were conducted with APPLE School teachers in the spring of 2009 (ten focus groups, *n* = 45) to examine the changes that had occurred as a result of the project, perceptions of advantages and disadvantages of being part of the project, strategies for implementation, and issues affecting sustainability and are described in two publications (DS3) [21, 22]. Semi-structured interviews were conducted with APPLE School principals (*n* = 29) (DS4) and HSHF principals (*n* = 17) (DS5) in the spring of 2013. Interviews focused on understanding the principal’s role within project implementation, facilitators and barriers, as well as the perceived culture change as a result of the project and have been described previously [17]. Additional details regarding the specific methods for each dataset including the setting, recruitment, sample characteristics, data generation and analysis protocols, saturation, and rigour can be found in the above publications [17, 21–24]. In sum, the present study is a secondary analysis of primary data from five separate datasets that includes 34 interviews with SHFs, focus groups with 45 teachers, and interviews with 46 principals. All participants provided informed written consent at the time of primary data collection which included consent for the present research, and all studies received ethical approval from the Health Research Ethics Board at the University of Alberta.

### Secondary data analysis

Interview data from all datasets were re-analyzed through a process of inductive content analysis following the stages outlined by Miles and Huberman [31] using NVivo 10.0 (QSR International, 2012) to describe the essential conditions of successful CSH implementation. While the first dataset included structured interviews, the transcribed data were treated the same as all other datasets. Given the nature of the structured interviews, the questions were open-ended and therefore warranted inclusion in the secondary data analysis. Meaningful segments of information were categorized using codes that emerged from the data. Thereafter, interpretive analysis was used to refine and collapse the data into larger categories, using a comparative technique [32], ensuring each category was unique, self-contained, and meaningful. This process allowed for existing categories to be refined and new categories to be generated. Ultimately, this analysis explored the facilitators and barriers of CSH implementation from the perspective of teachers, principals, and SHF. These factors, in turn, helped to inform the development and operationalization of the essential conditions necessary for successful CSH implementation. Three members of the research team (KS, GM, ER) met regularly to discuss emerging themes and refine the results. Peer debriefing (KS, GM, JO, PV, ER) served as a strategy to ensure rigour through examination of potential bias and by ensuring the researchers were both responsive and reflexive. Member checking with key project stakeholders (JF, MS, EW) also helped to further refine the results and add to the trustworthiness in the data.

## Results

**Fig. 1:**
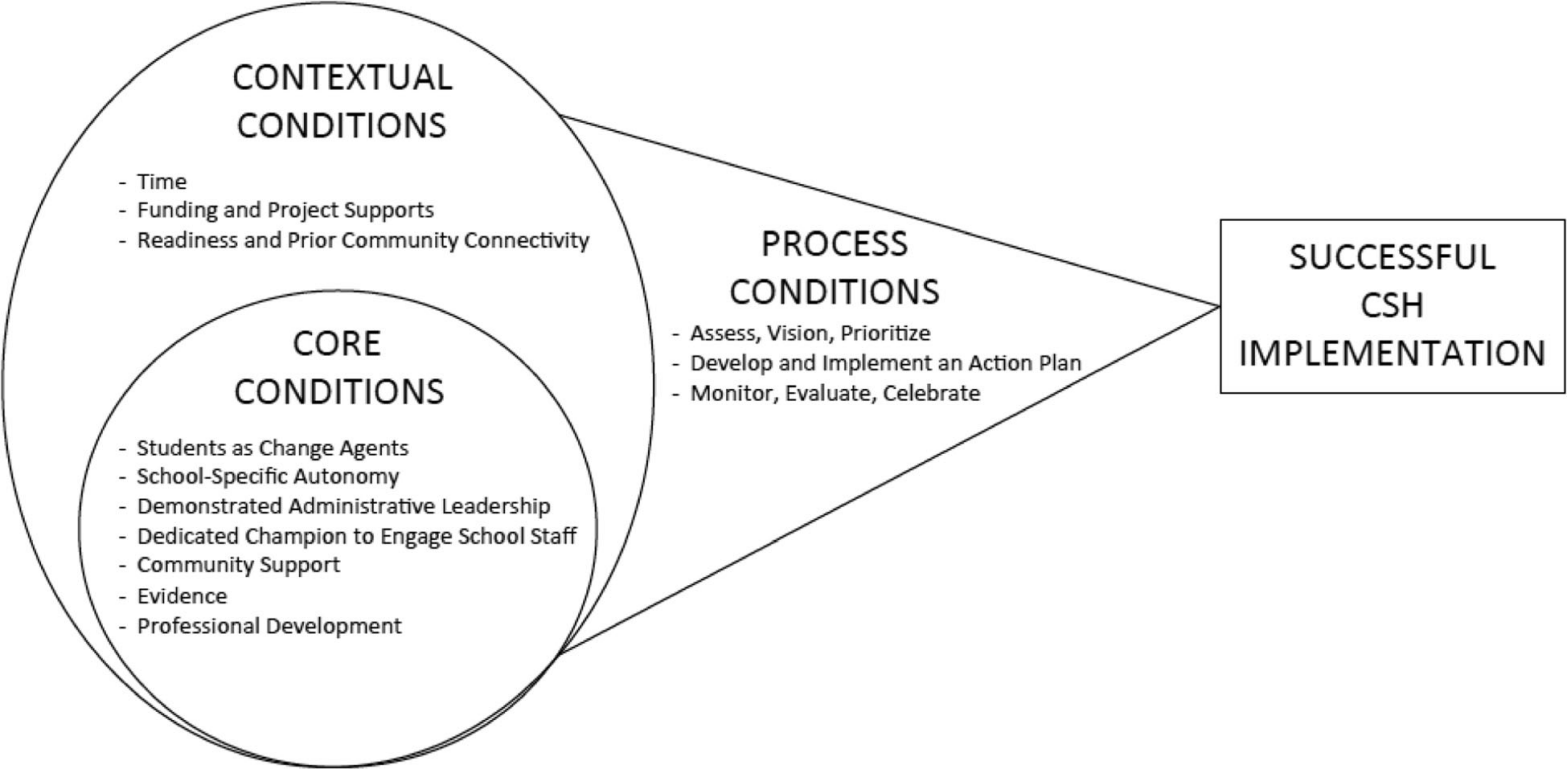
Essential Conditions for Successful Implementation of CSH

Inductive content analysis revealed many themes, here called conditions, that were divided into two categories: ‘core conditions’ which were conditions necessary for CSH to be successfully implemented and were thus at the ‘core’ and ‘contextual conditions’ which were not as essential but had a great degree of influence on the ability for the core conditions to be obtained. Themes for both core and contextual conditions are described in more detail below. As well, while not described as themes, it was the ‘process conditions’ (1. assess, vision and prioritize; 2. develop and implement an action plan; and 3. monitor, evaluate and celebrate) developed as part of APPLE Schools best practice that allowed for the ‘core’ and the ‘contextual conditions’ to be mobilized in practice to facilitate CSH implementation. Collectively, these ‘process conditions’ previously established by APPLE Schools and the presently described ‘core’ and ‘contextual conditions’ represent the essential conditions for successful implementation of CSH (illustrated in Fig. 1).

### Core conditions

Factors identified within this theme were emphasized as core conditions for CSH implementation across all stakeholders. In other words, without these conditions, participants felt CSH could not be successfully implemented. These conditions included: students as change agents; school-specific autonomy; demonstrated administrative leadership; dedicated champion to engage school staff; community support; evidence; and professional development.

#### Students as change agents

All stakeholders believed that students were the heart of the project and were the reason for wanting to implement CSH. As one teacher noted: *“…I think we look at it as we want our students to do better academically, socially, emotionally and everything that the APPLE School project does supports that.”* (DS3). Participants also reported that students who were enthusiastic and energized by the project were more likely to accept and engage within the project, and to communicate the CSH message beyond the school walls, propelling the project forward. As outlined by one SHF: *“[You are] not going to do a whole bunch of stuff in the school to try to affect kids’ health if kids don’t like what you’re doing. That’s just a reality of it.”* (DS2).

Notably, students were seen as the drivers of change in the home environment. While parental support was seen as a facilitator of CSH implementation, it was often difficult to engage the parent group. Stakeholders suggested that a potential means of engaging parents was through the buy-in of the children. As stated by one SHF: *“I’m hoping that through the students that they’ll get the parents…And then obviously those parents that are more involved…can model it for the kids themselves.”* (DS2). As a result, most stakeholder groups felt that initially it was best to ensure parents were aware of the changes, but to continue focusing on the children. Once engaged, parents were reported to communicate the CSH message more broadly, helping to reinforce the cultural shift. One principal summarized this phenomenon: *“…if the parents are on board…they can be great advocates for the things that we [want to] change in the bigger community because they do the parking lot talk….”* (DS4). In sum, while the engagement of both students and their families should remain a priority, participants felt that students played a much bigger role in influencing the home environment and therefore prioritized their involvement in the project.

#### School-specific autonomy

A core condition identified by stakeholders was the autonomy of each school, which required customization of the intervention to meet local needs. Autonomy was seen as imperative to build a sense of ownership for each school community. The intervention needed to be flexible to allow each school to build upon their strengths, assets and needs. As one teacher indicated: *“Not every school is going to have the same way of doing things [or have] the same issues and needs…so if you can be flexible enough and respond to the things that are happening in the school, you’re in. I think the project is more beneficial that way.”* (DS3). Participants felt that the process conditions as well as their ability to use school-specific evidence from evaluation reports were the drivers of this core condition. Action plans were developed through consultation with students, parents, teachers, school staff, and principals, as well as through the creation of an APPLE Core or Wellness Committee within the school which included community representation. Evidence provided through evaluation also allowed each school to customize the intervention based on data-driven decisions unique to their school community. One incredible success story is illustrated by an administrator who noted: *“47 % of our families reported that they were worried that food would run out before more money would come in. As a result, policy was changed immediately and all field trips and hot lunches became free.”* (DS5). It should be noted that securing funding is often quite difficult, but in this case the local community responded to such powerful evidence.

#### Demonstrated administrative leadership

All stakeholders strongly emphasized the importance of demonstrated leadership for successful CSH implementation, particularly the role of the school principal. This finding was perhaps the strongest result to emerge from the secondary analysis. Demonstrated administrative leadership differed from passive buy-in from the principal in that the principal was seen to play an invaluable role throughout the process of implementing CSH, and a key stakeholder in truly being able to facilitate a culture shift within a school community. The principal was seen as an active member of the implementation team and not purely a strong supporter of other champions such as the SHF. Teachers indicated that active leadership by the school principal allowed for CSH to become an essential component of the school’s agenda and thus was an identified priority area, as emphasized by one teacher: *“…I think if you’re in a school that didn't have an administrator who felt that healthy, a healthy lifestyle was important the program wouldn’t be what it is.”* (DS3). SHFs also emphasized the vital role that the school principal played as being a powerful influence for what practices are adopted and maintained within the school. As quoted by one SHF: *“I think the principal is probably the most important. Because anything that goes on has to go through him and if he’s not supportive, then it’s not going to happen.”* (DS2). One principal summarized their role by stating: *“…it’s a huge role because you really set the vision…everybody is watching you for the leadership so you have to choose carefully the direction that you want to head.”* (DS5). Principals were seen as key advocates and role models for CSH, as one administrator mentioned: “*I truly believe that I have to walk the talk…Kids won’t listen to what we say, they’ll listen to what they see us doing*” (DS4).

#### Dedicated champion to engage school staff

While the role of the administrator was seen as imperative from a leadership perspective, participants also indicated that having a SHF, or school health champion, was imperative to get the project up and running. Teachers, in particular, viewed the role of the SHF as a catalyst for the ongoing integration of the CSH model into daily practices, and this presence kept them focused on the project. Principals felt that without the champion, the project would not have been initiated due to the busy nature of the school environment. Although an important factor, the SHF was also mentioned as a barrier by some, particularly within the latter stages of implementation. As implementation proceeded, others in the school may have become apathetic in picking up the tasks of the SHF, limiting the sustainability of the project. One principal spoke to this problem in the following quote: *“… when you designate somebody to have a particular job in one area…you celebrate the fact that you’ve got somebody in charge that’s going to spearhead that. But you risk losing everybody else because they now say ‘but she’s getting paid to do that organization’s stuff. So why should I take that on and volunteer my extra hours…?’”* (DS4).

It was reported by participants that in order to truly shift a school culture, all members of the school community needed to play a role. SHFs identified that learning to go from ‘doing’ to ‘facilitating’ in the school was essential for sustainability of successful changes in the school culture. SHFs reported that all school staff, and notably teachers were seen as a core component of CSH implementation in that they are the ones who are actively integrating CSH into practice at a ground-level. The need for school staff buy-in was also supported by the principal: *“…it has got to filter down to the individual classroom teacher, to the secretary, to the custodians, to the educational assistants – they’re the ones who are, you know, in the trenches and they’re having to actually implement these things.”* (DS5). SHFs echoed this sentiment, by alluding to the role of a teacher as not only an advocate, but also a gatekeeper of the project, with one SHF stating: *“You need buy-in from the teachers… if your teachers don’t believe in it, then they’re not going to promote it within their classrooms, even though their administrator has an expectation of them.”* (DS1). While the role of the teacher actively advocating for CSH took time to evolve and was not as prominent during the initial stages of implementation, their role flourished over time as implementation proceeded and as the project became one that was led by distributed leadership.

#### Community support

All stakeholders mentioned the key role that both internal and external partnerships played in alleviating the pressures of implementation and essentially created a ‘village’ of those supporting CSH. Participants indicated that the internal relationships formed between school staff, SHFs, administrators and other project staff was invaluable to share lessons shared. As one SHF stated in regards to support from other SHFs: *“I think if you didn’t have that, you could feel so isolated.”* (DS1). When examining external supports, one principal asserted: *“I don’t think you can do it by yourself. There are too many needs at these schools and so you have to be able to bring in outside partnerships.”* (DS4). SHF also appreciated knowing that there were external resources to support their work.

While participants stressed that the majority of their time during the initial stages of implementation needed to be spent engaging those internal to the school community, they did indicate that having strong community connections strengthened the type of programs the schools could offer and enhanced the social environment. As stated by one SHF: “*Cooperation. And relationships for sure. Starting within the school and then getting community. Definitely, like – I think unless you have the cooperation from everyone involved in the school community, I mean then it’s going to be a struggle.”* (DS2). Because stakeholders felt it was important to spend time building relationships with internal stakeholders during the initial implementation of CSH, it was seen to be beneficial to begin establishing connections with external community partners and services prior to implementation.

#### Evidence

The ability to use local school-level data (both process and outcome) was seen as essential for planning, refining, and supporting the implementation of CSH. This was in the form of both research findings relating to health behaviours and environmental-level changes, as well as more informal evaluations. In regards to planning, stakeholders indicated that the research evidence in the form of individualized school reports allowed them to make decisions based on their school context. These reports include information on students’ physical activity, nutrition, screen time, sleep habits, BMI, as well as their home and school environments. These reports are provided by the University of Alberta’s School of Public Health following annual data collection. One principal compared the use of their local data to the use of standardized test results: *“We analyze the data, we say – it’s like our provincial achievement test results. What’s the data telling us? How is that going to inform our planning for next year?”* (DS5).

In addition to planning, stakeholders indicated data were also useful to support the intervention and increased buy-in from the school community, including parents, staff, and the school district. As indicated by one SHF: “…*when I meet with parents and staff here and there, they’re like, ‘Well do you have evidence? Do you have proof?’, ‘Do you have proof that it helps kids behave better, stay focused in class?’ Yeah. I do…cause then you’re going to get so much more buy-in.”* (DS2). As well, one principal stated: *“the information is shared with staff and it goes into our priorities for why we need to continue…and certainly the information is shared with my school council.”* (DS5). As well, the evidence allowed each school community to not only celebrate their successes but also to adapt the intervention on the basis of findings.

#### Professional development

Stakeholders believed that both initial and ongoing professional development were paramount in informing school members of the project goals, objectives, and rationale , and built self-efficacy for project implementation. SHFs specifically stated that the training strengthened their knowledge and essential skills for working in the schools and built not only their competence but also their confidence in implementing CSH. As quoted by one: *“I think we have to have meaningful professional development for us as facilitators and also for staff.”* (DS1). As well, another SHF stated *“Seeing practical examples in action was essential to prepare me for the work.”* (DS1). Teachers appreciated the resources and professional development provided through staff meetings, the SHFs and other professionals, allowing project material to become infused into the classroom. This in turn increased teacher ownership and support for the project. One teacher commented regarding the training and education provided by the SHF: *“…she’s teaching your kids but she’s also teaching you, so then you’re going to have that knowledge and then carry it forward when she’s not here.”* (DS3). As well, principals indicated that without the professional development prior to and throughout implementation they would not have had a clear understanding of the project, which they felt was necessary to ensure the project values and their values were aligned prior to implementation.

### Contextual conditions

Contextual conditions were cited to have a great degree of influence on the ability for the core conditions, mentioned above, to be obtained. As such, they acted as important considerations for successful CSH implementation and included: time; funding and project supports; and readiness and prior community connectivity.

#### Time

All stakeholders emphasized the role that time plays within the implementation process. Enough time was needed to be dedicated to the project in order for it to be successful. This was often difficult within a busy school environment where multiple priorities and competing interests often interfered with project objectives. As one principal indicated: *“…the challenge is that the central purpose of the school is teaching and learning and working towards building students’ achievement. And there is only a certain amount of time built into the school calendar. And realistically, that’s what teachers have to focus on.”* (DS4). Time was also cited as essential in order to prevent CSH from being viewed as an add-on, but rather as an embedded part of the school’s culture. As suggested by one SHF: *“So being able to give time to people to dedicate to health promoting schools and not just have it be their passion that they do on the side. I think that’s pretty essential.”* (DS2). Principals also emphasized that allotted time allowed for implementation to become more impactful. As stated by one principal: “*You can get all the money in the world but if you don’t get an extra two hours a day to implement, you’re not going to you know, impact something.”* (DS5).

#### Funding and project supports

Stakeholders asserted that the financial support from the project greatly facilitated implementation. As stated by one principal: *“The financial support from the project was huge…it’s not about questioning whether the healthy initiatives are important. It’s about our primary role is to be teachers and leaders of education…”* (DS4). Stakeholders recognized that school budgets were tight, requiring external resources to support CSH objectives that lay outside of traditional school priorities. This sentiment was relayed by another principal: *“School resources are strained. Certainly it’s difficult to justify taking educational dollars away from student to learn and putting that towards a lunch program.”*(DS5). The structure and managerial supports provided through the CSH projects also facilitated implementation. This included support from CSH team managers and project staff who were actively involved in ensuring schools were accountable and had the help and guidance they needed during implementation, including grant writing to secure sustainable funding after initial implementation.

#### Readiness and prior community connectivity

It was important for stakeholders to have an understanding for CSH and the reason for its existence as this knowledge helped to build competency and increased ownership and enthusiasm over the project. For example, once teachers were able to develop a clear understanding of the project’s objectives, implementation was viewed as *“natural”* and healthy eating and active living activities were *“easily incorporated.”* It was also important for stakeholders to feel comfortable within their school, with foundational knowledge of the context and resources as well as established relationships in the school. As expressed by one principal: *“…I think the biggest challenge, again, for me, was I don’t think an APPLE school should go into a place where the principal is brand new… when you come in new like that, it takes you six months…as the principal…to figure out your people.”* (DS4). SHFs new to a school found that they spent a great deal of time in the beginning building trusting relationships with others. This was expressed by one SHF who shared: *“I think the biggest thing is you have to, you know, develop that relationship. You have to meet everyone, let them know who you are…”* (DS1).

## Discussion

This study is the first to comprehensively examine and operationalize the essential conditions of CSH implementation. Previous studies have attempted to characterize the essential conditions of CSH through a more theoretical perspective of conditions believed to be important for implementation [14, 16, 33]. However the work presented here is unique in its inclusion of multiple data sources, including first-hand accounts of a multitude of CSH stakeholders, particularly those working on the ground within successful CSH projects, as evidenced by a culture shift [17, 21–24] and improvements in health behaviours [9, 18]. Collectively, we reported core, contextual, and process conditions that together form the essential conditions of successful implementation of CSH. While the process conditions developed by APPLE Schools have been previously reported and discussed [19, 20, 27], the subsequent discussion sections will further examine the core and contextual conditions in relation to the current body of literature.

### Core conditions

Our study adds to the literature by operationalizing, through the core conditions, the role of key stakeholders in facilitating a shift in school culture. A core component of CSH has been identified as student engagement [34, 35]. This aspect meets a basic tenet of health promotion, namely the involvement of the target group in the development and delivery of an intervention. Furthermore, parents have been identified as key partners in the planning and implementation of CSH [12]. Our study revealed that notably students were great advocates at extending the CSH message beyond the school, and were the drivers of change at home. While this phenomenon has been briefly discussed in the literature [36, 37] , our study adds further support to this practice as a means to facilitate the sustainability of such initiatives. Of note, members of our research team recently examined how children translate school-learned behaviours home. Results indicated students were indeed drivers of change in the home environment and were successful at both communicating healthy messages to families as well as changing families’ health behaviors (McKernan C, Chahal H, Gleddie D, Montemurro G, Veugelers P, Storey K: Comprehensive school health and achieving change in the home environment: student insights from a photovoice project, in review).

In order for schools to become involved in a CSH project, it is important that they feel a sense of autonomy regarding the CSH project. It has been suggested that school stakeholders may feel threatened by imposed goals and practices [38], and that it is therefore vital to build shared visions and directions in school health promotion [39] as well as to tailor programs to individual schools’ needs while also aligning the intervention with schools’ core aims [40]. Further, anchoring initiatives within the school through goal creation, and matching these goals with those guiding the overall school is deemed essential [41] and is stimulated through the establishment of shared values and beliefs [42]. While many CSH initiatives are mindful of project autonomy, others have taken a more ‘one size fits all approach’. The present findings support the principles of health promotion [43] and serve as a reminder that each school community has unique assets, strengths and needs.

In regards to CSH implementation, the support of the principal seems to be emphasized within the literature, a component for which our group has previously examined in detail and can attest to [17]. This is echoed in Samdal & Rowling’s [14] review with several sources of evidence [41, 44, 45] highlighting the importance of leadership in order to achieve successful implementation. The present findings are unique in that our study suggests that in order for CSH implementation to be successful to shift the school culture, the principal must be an active member of the implementation team and not purely a passive supporter of other school health champions. Aside from having central support from the school leader, the concept of distributed leadership [46] has been emphasized within the literature, particularly in settings-based approaches to health. Here, participation and ownership is encouraged on behalf of the wider school community [33]. This also highlights the importance of teacher and staff support and involvement for the initiative, another important core condition revealed in the present analysis as well as in Langford et al.’s review [40]. Previous research has found that principals perceived teachers’ competence and understanding as an invaluable component of CSH, whereby motivated teachers were central to the perpetuation and maintenance of the initiative [47]. This sentiment was reiterated in the present analysis, as emphasized from the viewpoints of several school stakeholders, not just the principal.

Within the present study, the SHF was seen as key for implementation of CSH, so long as their role evolved from that of ‘doing’ to that of ‘facilitating’ and ensuring the project was led through distributed leadership to ensure sustainability. Others have suggested that the SHF, or champion, is the most important human resource within the school when it comes to project implementation [16], but there is limited discussion in the literature to the role of the SHF in the face of project sustainability. Studies by Christian et al. [48] and Clarke et al. [49] have highlighted that among the challenges schools and teachers face in implementing health interventions, are academic priorities and a lack of staff support. Thus having dedicated staff in the form of SHFs may help to address this point so as the champion facilitates engagement of staff to achieve buy-in and is not the sole individual responsible for implementation. Paired with our findings this opens a space for discussion surrounding this point, and further investigation is necessary to fully delineate the influence of such an individual on implementation and sustainability of CSH.

Within Samdal & Rowling’s [14] review, the importance of establishing partnerships and networking was identified as one component to develop a health promoting school [15, 41, 44, 50]. Our study also emphasizes the importance of schools developing and maintaining partnerships with outside community organizations so the support of a ‘village’ is realized when implementing CSH. The specific make-up of these partnerships and how they came to be established remains less clear, and is suggested as a further area of exploration to assist schools in better navigating CSH implementation. As captured by headteachers in a study by Howard-Drake et al. [51], external partnerships (e.g., with school nurses, health specialists, and private companies), may be advantageous in alleviating a school’s lack of internal capacity for school-based health promotion. A notable finding of the present analysis was the need to focus on internal partnerships during the initial stages of implementation. This may seem to contradict traditional health promotion practices [43] and is possibly a unique concept when working within school settings.

As a research project, both APPLE Schools and HSHF received school-specific data to inform practice. Participants reported that they were keen to share evaluation data with their school community and ensured that they remain engaged within the process and celebrated each milestone and accomplishment. Data sharing has previously been shown to increase stakeholder engagement [16], by stimulating collective reflection and providing evidence of progress, which becomes a significant source of motivation [52]. Furthermore, school involvement and connectivity to the CSH initiative are facilitated through continuous communication as well as the celebration of successes as they happen [16]. These practices also ensure project sustainability [53]. Although using evidence to influence practice is discussed in some of the literature, we feel that the present study reaffirms its importance, especially considering its lack of inclusion within Samdal & Rowling’s [14] review.

While it is important to have engaged involvement from all school community stakeholders, it is essential that all stakeholders are informed and educated on CSH implementation processes, and thus both initial and ongoing professional development is a core condition of implementation. In Canada, although recommended, CSH training is not included within the formal education of teachers [12, 23]. Professional development opportunities therefore become ever-more important to foster good health education practices in schools [10, 14, 40, 41, 44, 45].

### Contextual conditions

The contextual conditions revealed in this analysis included time, funding and project supports, as well as readiness and prior community connectivity. Within the present analysis, the contextual condition of ‘time’ referred to the need for the project to receive enough attention and prioritization in the busy school environment as to not to be seen as an add-on, but rather as an integral piece of school operations. Stolp, Wilkins, & Raine [16] emphasized the importance of time for sharing experiences and information exchange between schools as a facilitator of healthy school community development, a phenomenon that is more generally discussed in the literature [14, 54]. Taking the implementation process slowly, as to avoid overwhelming the school community and increase the likelihood of a positive impact, is also highlighted in the literature [12, 55].

Surprisingly, ‘funding and project support’ is not discussed at length with the CSH implementation literature. Deschesnes, Martin, & Jomphe Hill [15] suggest that the political and financial support from decision makers is required for adequate implementation of comprehensive approaches. Others have also mentioned the lack of such support as a significant barrier to implementation [56]. Financial and project support has also been linked to other conditions of implementation including garnering the proper resources, training, and available time [57]. Again, our study is the first to explicitly outline funding and project support as an essential condition of CSH implementation.

The need for stakeholders to have an understanding of their school context and pre-established trusting relationships was also emphasized in the present study, particularly prior to commencing implementation through the contextual condition of ‘ readiness and prior community connectivity’. We believe that our study is the first to emphasize this in the context of CSH implementation. Others have emphasized the need for strong relationships to be developed between all stakeholders [16], but we are not aware of any who have framed this in terms of a necessity and essential condition of CSH implementation.

As discussed, contextual conditions are intimately tied to the core conditions of implementation. Without the time, money, or social connectivity and contextual awareness, simply having trained staff and school personnel on-board with the initiative would certainly not be enough for implementation to be successful. This again, is similar to the relational and organizational support context as outlined by Samdal & Rowling [14], which in some ways brings together all other necessary components for CSH implementation.

In order to demonstrate value in the investment of CSH, we need to advance and improve our research methods for evaluating CSH implementation [40], which could be guided by the essential conditions discussed above. Not only will this increase our understanding of the effectiveness of CSH, but will be transferable to other areas where complex thinking is needed to tackle health problems such as obesity, chronic disease, tobacco control, and communicable disease. New knowledge will be used to strengthen and optimize CSH implementation, and ultimately will inform and direct new public policy in order to prevent chronic diseases and improve the health of children and youth.

### Strengths and limitations

As this study had a qualitative focus, results are not necessarily generalizable to all CSH studies. Further, secondary data analysis is limited by researcher presence during primary data collection. Primary researchers will always have a privileged relationship with the data generated. An additional limitation of the primary data is the potential for bias due to self-selection of participants, whereas only those interested or supportive of the project participated in the research. Despite this, CSH interventions were applied across different contexts and interview data was gathered across different degrees and stages of implementation, emphasizing the transferability of the present findings. A strength of the present study is its combination of multiple data sources as a means of triangulation [30]. By triangulating the data through multiple stakeholder interviews we have attempted to maximize the credibility of our findings, while minimizing the impact of potential biases [58]. Another overall strength of this research includes the fact that APPLE Schools has been shown to be an effective intervention at shifting the school culture [17, 21–24] and targeting healthy eating and physical activity, as well as reducing health inequalities [9, 18]. Thus, not only has this study provided a detailed depiction of the essential conditions of CSH implementation, but has done so using data from a highly effective CSH project. It is thus hoped that these findings be applied in different settings to bring about the optimization of CSH project implementation, leading to a broader positive impact on the health and wellbeing of children and youth.

## Conclusions

In addition to previously established process conditions, this study revealed core and contextual conditions that ultimately provides a set of essential conditions for CSH implementation to shift a school culture and improve health behaviours in children. While many of the aforementioned conditions have been touched upon within the existing literature, this study reaffirms their categorization as essential conditions of CSH implementation, creating a stronger evidence-base of their importance. Overall, more research is needed to both test the applicability of the identified essential conditions more broadly. It is our intention to provide further confirmation of these factors by interviewing stakeholders throughout Canada to provide further evidence to support/disprove any of the conditions mentioned. Although further investigation may be required to confirm this, we believe that school communities should be encouraged to utilize the essential conditions of CSH outlined in this study. This detail will provide schools with a set of understandable and tangible guidelines to facilitate implementation of CSH. We believe that the present research greatly contributes to the evidence-base of CSH implementation, ultimately helping to shape its optimization. Such research is important as it helps bolster the CSH framework that has been shown to improve the education, health, and well-being of school-aged children [15].

## Abbreviations

APPLE Schools: Alberta project promoting active living and healthy eating in schools
CSH: Comprehensive school health
DS: Dataset
HSHF: Healthy schools–healthy future
JCSH: Joint consortium for school health
SHF: School health facilitators
